# Colonisation and Transmission Dynamics of *Candida auris* among Chronic Respiratory Diseases Patients Hospitalised in a Chest Hospital, Delhi, India: A Comparative Analysis of Whole Genome Sequencing and Microsatellite Typing

**DOI:** 10.3390/jof7020081

**Published:** 2021-01-26

**Authors:** Anamika Yadav, Anubhav Singh, Yue Wang, Merlijn HI van Haren, Ashutosh Singh, Theun de Groot, Jacques F. Meis, Jianping Xu, Anuradha Chowdhary

**Affiliations:** 1 Department of Medical Mycology, Vallabhbhai Patel Chest Institute, University of Delhi, Delhi 110007, India; yadavanamika761995@gmail.com (A.Y.); dr2.anubhavsingh@gmail.com (A.S.); singh90.ashutosh@gmail.com (A.S.); 2 Department of Zoology, Ramjas College, University of Delhi, Delhi 110007, India; 3 Department of Biology, McMaster University, Hamilton, ON L8S 4K1, Canada; wangy660@mcmaster.ca (Y.W.); jpxu@mcmaster.ca (J.X.); 4 Department of Medical Microbiology and Infectious Diseases, Canisius-Wilhelmina Hospital, 6532 SZ Nijmegen, The Netherlands; merlijnvanharen@gmail.com (M.H.v.H.); T.deGroot@cwz.nl (T.d.G.); jacques.meis@gmail.com (J.F.M.); 5 Centre of Expertise in Mycology Radboudumc/CWZ, 6532 SZ Nijmegen, The Netherlands

**Keywords:** *Candida auris*, colonisation, microsatellite typing, whole genome sequencing, *ERG11*, *TAC1B*, amphotericin B resistance in *C. auris*, India

## Abstract

*Candida auris* is a nosocomial pathogen responsible for an expanding global public health threat. This ascomycete yeast has been frequently isolated from hospital environments, representing a significant reservoir for transmission in healthcare settings. Here, we investigated the relationships among *C. auris* isolates from patients with chronic respiratory diseases admitted in a chest hospital and from their fomites, using whole-genome sequencing (WGS) and multilocus microsatellite genotyping. Overall, 37.5% (*n* = 12/32) patients developed colonisation by *C. auris* including 9.3% of the screened patients that were colonised at the time of admission and 75% remained colonised till discharge. Furthermore, 10% of fomite samples contained *C. auris* in rooms about 8.5 days after *C. auris* colonised patients were admitted. WGS and microsatellite typing revealed that multiple strains contaminated the fomites and colonised different body sites of patients. Notably, 37% of *C. auris* isolates were resistant to amphotericin B but with no amino acid substitution in *ERG2*, *ERG3*, *ERG5*, and *ERG6* as compared to the reference strain B8441 in any of our strains. In addition, 55% of *C. auris* isolates likely had two copies of the *MDR1* gene. Our results suggest significant genetic and ecological diversities of *C. auris* in healthcare setting. The WGS and microsatellite genotyping methods provided complementary results in genotype identification.

## 1. Introduction

*Candida auris* is a multidrug-resistant fungus that has been implicated in hospital-associated outbreaks of life-threatening invasive infections with high mortality. Since its first report in 2009, *C. auris* has caused major outbreaks in healthcare facilities in Colombia, India, Kenya, Kuwait, Oman, Pakistan, South Africa, Spain, United Kingdom, United States, and Venezuela [1,2,3,4,5,6,7,8,9,10,11,12,13]. The predisposition of *C. auris* to colonise hospitalised patients and its potential for person-to-person spread from colonised and infected patients have raised concerns about further large-scale outbreaks in healthcare facilities [14] The other major concern in the nosocomial transmission is the propensity of shedding viable yeast cells from patients, thus contaminating environmental surfaces in rooms of colonised or infected patients. Remarkably, *C. auris* has been frequently recovered from common shared surfaces in the hospital environment, suggesting the potential risk of environmental transmission in healthcare settings [14,15]. Indeed, molecular epidemiology investigation has provided key insights into the origin and transmission of *C. auris*. However, the dynamics of transmission of *C. auris* in hospital settings using whole genome sequencing (WGS) have been analysed in limited studies, primarily from Colombia, the United Kingdom, and United States [10,11,14,16,17]. Specifically, WGS of *C. auris* isolates from patient contacts, healthcare workers, and the environment in 4 hospitals with *C. auris* outbreaks in Colombia revealed widespread environmental contamination and colonisation among patients and healthcare workers. Their study reported genetically identical isolates within hospitals that connected patients to environmental surfaces and healthcare workers [14]. In contrast, the genetic epidemiology of an outbreak of *C. auris* in a specialized cardiothoracic London hospital using MinION nanopore sequencing technology suggested that patients were infected with a genetically heterogeneous population [16]. Further, none of the patients and the hospital environment was infected with a single, clonally propagating *C. auris* strain. Interestingly, a *C. auris* outbreak in the neurosciences intensive care unit (ICU) of the Oxford University Hospital, United Kingdom was linked to reusable axillary temperature probes. WGS identified that isolates from reusable equipment were genetically related to patient isolates [11].

In this study, we aimed to investigate the genetic diversity and transmission dynamics of *C. auris,* colonising the patients with chronic respiratory diseases admitted in a referral chest hospital in Delhi, India and assessed the environmental contamination in rooms occupied by colonised patients. To understand the mode of spread between patients within the hospital, we performed WGS and evaluated the recently described microsatellite length polymorphism (MLP) typing using a short tandem repeats (STRs) assay for *C. auris* in the transmission of this yeast in clinical settings [18].

## 2. Methods

### 2.1. Ethical Statement

The study was approved by Vallabhbhai Patel Chest Institute (2019/2401).

#### Study Design

Patients with chronic respiratory diseases admitted to the medical ward during the six months period (December 2019–May 2020), were enrolled for screening of *Candida auris*. The study was conducted in 16 rooms of a medical ward housing 64 beds, primarily for patients with respiratory disorders, admitted for acute episodes or exacerbations of chronic diseases. Patients were screened at the day of hospitalisation by swabbing 4 sites (ear, nose, axilla, groin) individually for each patient. Blood cultures were collected from patients whose screening swabs yielded *C. auris*. Swab samples were repeated every week up to the day of discharge of the patients. Environmental sampling of all rooms with colonised patients was conducted at the time of patient contact and repeated every week when patients were sampled till discharge.

### 2.2. Collection and Processing of Clinical and Environmental Specimens

#### 2.2.1. Collection of Swab Specimens

(i) Skin swabs: The swabs were collected individually from axilla, groin, nose, and ear of each patient using sterile cotton swabs premoistened in sterile physiological saline. Swabs were placed in capped propylene tubes (HIMEDIA, Mumbai, India) and were transported to the laboratory within an hour at ambient temperature.

(ii) Environmental swabs: The premoistened swabs were swept back and forth over an area of 25 cm for effective collection of samples [19]. Environment samples were collected from different sites near each patient’s bed including bed railing, bed sheet, pillow, bedside trolly, floor, and air conditioner air wings. Surface swabs were also collected from medical equipment (thermometer, B.P. cuffs, ECG clip and sucker, oxygen mask, and nebuliser) and portable devices (mobile, wheelchair, and intravenous pole). Further, random surface samples were collected from water faucets and sinks present in each ward. For each site, two repeat swabs were collected.

#### 2.2.2. Processing of Swab Specimens

Of the two swabs from each site, one was inoculated onto nonselective media i.e., Sabouraud dextrose agar (SDA) containing chloramphenicol (25 mg/L) and gentamicin (40 mg/L) at 37 °C for 48–72 h. All yeast colonies that grew on SDA were purified and identified. The other swab was immersed in selective yeast nitrogen enrichment broth (YNB) containing 10% NaCl and 2% mannitol as a carbon source and vortexed and incubated at 37 °C for 72–96 h [20]. 400 µL of the broth was inoculated on one set each of CHROMagar^TM^ *Candida* Plus agar [21], a differential medium for *C. auris* and CHROMagar^TM^ *Candida* medium for 48 h at 37 °C. After 48 h, all colonies that grew on both CHROMagar plates were sub-cultured on SDA plates and incubated for 24 h at 37 °C for purification and identification.

#### 2.2.3. Yeast Identification and Antifungal Susceptibility Testing

All yeast isolates were identified by MALDI TOF-MS (Bruker Biotyper OC version 3.1, Daltonics, Bremen, Germany) with a score value of >2 against the Bruker’s database and in-house *C. auris* database [22]. Yeast isolates not identified by MALDI TOF-MS were identified by amplification and sequencing of the internal transcribed spacer (ITS) regions of the ribosomal DNA (rDNA) and the D1/D2 domain of the large subunit rDNA (28S) followed by GenBank basic local alignment search tool (BLAST) pairwise sequence alignment (http://www.ncbi.nlm.nih.gov/BLAST/Blast.cgi) [3].

Antifungal susceptibility testing (AFST) was performed using the CLSI broth microdilution method (BMD), following M38-Ed3 [23]. The antifungals tested were fluconazole (FLU, Sigma, St Louis, MO, USA), itraconazole (ITC, Lee Pharma, Hyderabad, India), voriconazole (VRC, Pfizer, Groton, CT, USA), posaconazole (POS, Merck, Whitehouse Station, NJ, USA), isavuconazole (ISA, Basilea Pharmaceutical, Basel, Switzerland), 5-flucytosine (5-FC, Sigma), caspofungin (CFG, Merck), micafungin (MFG, Astellas, Toyama, Japan), anidulafungin (AFG, Pfizer) and amphotericin B (AMB, Sigma). The drugs were tested for 10 (two-fold) dilutions and the drug concentration ranges were: FLU, 0.25–128 mg/L; ITC, VRC, and AMB, 0.03–16 mg/L; POS, ISA, AFG, MFG, CFG, 0.015–8 mg/L; 5-FC, 0.125–64 mg/L. *Candida krusei* strain ATCC 6258 and *Candida parapsilosis* strain ATCC 22019 were used as quality control strains. The modal MIC, geometric mean (GM) MIC with 95% CIs, MIC50, MIC90, median, and range were calculated using Prism version 6.00 (GraphPad Software). The MIC endpoints for all the drugs except amphotericin B were defined as the lowest drug concentration that caused a prominent decrease in growth (50%) in relation to the controls. For amphotericin B, the MIC was defined as the lowest concentration at which there was 100% inhibition of growth compared with the drug-free control wells.

#### 2.2.4. Genome Sequencing, SNP Calling, and Phylogenetic Analysis

For genomic sequencing, the genomic DNA from representative *C. auris* isolates were extracted using a column-based method with a QIAamp DNA minikit (Qiagen, Hilden, Germany) and quantified by QUBIT 3 Fluorometer using dS DNA HS Dye. WGS libraries were prepared using NEBNext ultra II DNA FS kit (New England Biolabs, Ipswich, MA, USA). In brief, 200 ng of genomic DNA was enzymatically fragmented by targeting 200–300 bp fragments sizes followed by purification using AMPure beads (Beckman Coulter life Sciences, Indianapolis, IN, USA). For sequencing, the libraries were normalized to 10 nMol/L concentrations and pooled together at equal volumes. Further, the library pools were denatured using freshly prepared 0.2 N NaOH for cluster generation on cBOT and sequenced on Illumina HiSeq 4000 [24].

Variant identification and phylogenetic analysis: *Candida auris* strain B8441 assembly V2 was obtained from NCBI (https://www.ncbi.nlm.nih.gov/genome/38761?genome_assembly_id=353836) and used as the reference clade I strain. Further, genome sequences of 18 previously published Indian *C. auris* strains (B11200, B11201, B11205-B11207, B11209, B11210, B11212-B11218, VPCI_510/P/14, VPCI_692/P/12, VPCI_550/P/14, VPCI_479/P/13) [25,26] were retrieved for comparison. Single nucleotide polymorphisms (SNPs) were identified using the NASP pipeline (Northern Arizona SNP Pipeline, http://tgennorth.github.io/NASP/). Reads were trimmed using Trimmomatic v0.39 [27] and aligned against the reference genome using BWA v0.7.17 [28]. SNP sites were identified using GATK v2.7.4 [29]. The SNP sites were filtered if they were located in the repetitive regions of the reference genome, had a coverage lower than 10 x, or with less than 90% variant allele calls. For the phylogenetic analysis, 1154 SNP sites were concatenated. The maximum likelihood tree was constructed using RAxML based on 1000 bootstrap replicates and the ASC_GTRCAT nucleotide substitution model. The phylogeny was visualized using an online tool iTOL (https://itol.embl.de/).

To estimate the divergence time of the most recent common ancestor for the *C. auris* isolates, the Bayesian phylogenies were constructed with BEAST v2.6.3 [30] under a general time reversible nucleotide substitution model, and a strict clock model with clock rate set as 1.0 × 10^−5^. Further, a coalescent exponential population model was applied. The tip dates were assigned according to the sampling dates and year midpoint was applied to samples without the month and date information. The Markov Chain Monte Carlo (MCMC) took 50,000,000 steps in the chain, and tree samples were logged every 1000 steps. Tracer v1.7.1 [31] was used to visualize and analyze the posterior MCMC samples. The effective sample size for each parameter was over 700, indicating that the MCMC runs had converged. The maximum clade credibility tree was created using TreeAnnotator v2.6.3 after discarding the first 10% as burn-in, and visualized using FigTree v1.4.4.

#### 2.2.5. Microsatellite Typing of *C. auris*

A single colony of *C. auris* was inoculated in 50 µL physiological saline (154 mM NaCl) and incubated for 5 min at 37 °C after addition of 200 U lyticase (Sigma-Aldrich, St. Louis, MO, USA). Subsequently, 450 µL physiological saline (154 mM NaCl) was added and the sample was incubated for 15 min at 100 °C and cooled down to room temperature. PCR amplification of the STR regions was performed on a thermocycler (Westburg-Biometra, Göttingen, Germany) using 1× Fast Start Taq polymerase buffer without MgCl_2_, dNTPs (0.2 mM), MgCl_2_ (3 mM), multiplex primers (0.2–0.8 µM), 1 U Faststart Taq polymerase (Roche Diagnostics, Germany), and DNA. A thermal protocol of 10 min denaturation at 95 °C, followed by 30 cycles, with 1 cycle consisting of 30 s denaturation at 95 °C, 30 s of annealing at 60 °C, and 1 min of extension at 72 °C, and a final incubation for 10 min at 72 °C. Subsequently, the PCR products were diluted 1:1000 in water, and 10 µL of diluent together with 0.12 µL orange 600 DNA size standard (NimaGen, Nijmegen, The Netherlands) was incubated for 1 min at 95 °C. The copy numbers of the 12 markers were determined for all isolates using GeneMapper software 5 (Applied Biosystems, Foster City, CA, USA). The size of the alleles was rounded. Relatedness between isolates was analysed using BioNumerics software version 7.6.1 (Applied Maths, Kortrijk, Belgium) via the unweighted pair group method with arithmetic averages (UPGMA) using the multistate categorical similarity coefficient. All markers were given an equal weight.

## 3. Results

### 3.1. Patient Details and C. auris Colonisation

During the six-month-period (December 2019–May 2020), a total of 195 patients were admitted. Of these, 32 patients who had a history of chronic respiratory diseases (>6 months duration) and required prolonged and/or repeated hospitalisation were screened. Of the 32 patients screened, 37.5% patients (*n* = 12, mean age 51 years; male = 11, female = 1) had or developed *C. auris* colonisation while hospitalised. These 12 *C. auris* colonised patients were admitted in seven rooms of the medical ward.

The clinical details and *C. auris* positivity at weekly intervals are detailed in Table 1. The majority of the colonised patients had chronic obstructive pulmonary diseases (COPD) along with post tuberculosis complications (*n* = 10). One patient each had HIV with pneumothorax and interstitial lung diseases (ILD) respectively (Table 1). Further, among the *C. auris* colonised cohort, 25% (*n* = 3) had underlying diabetes mellitus. Of the 12 patients colonised by *C. auris*, nine (75%) had a single site positive and three (25%) each had three sites positive. Further, the groin was the most frequently colonised site (75% *n* = 9) followed by nose (42%, *n* = 5) and ear (33%, *n* = 4). *C. auris* colonisation was detected in 25% (*n* = 3) of patients at the time of admission whereas the remaining patients (*n* = 9) developed colonisation after one week of admission. Overall, *C. auris* colonised patients (*n* = 12) were hospitalised for 10–150 days. Further, 75% (*n* = 9) of patients remained colonised till the day of discharge which ranged from 10 to 26 days whereas two patients had clearance of *C. auris* between 30 to 60 days. Further, three patients with *C. auris* colonisation were co-colonised with *C. albicans* (*n* = 2) and *C*. *tropicalis* (*n* = 1). In the remaining non-*auris* cohort of 20 patients, 14 were colonised by other yeasts and surveillance cultures in six patients remained negative for yeasts. *C. parapsilosis* and *C. albicans* represented the majority of non-*auris* cohort, including four patients each, followed by *C. orthopsilosis* and *C. guilliermondii* for a single patient each. Further, in three patients, *C. parapsilosis* showed co-colonisation with *C. orthopsilosis* in one; *C. tropicalis, C. albicans,* and *C. guilliermondii* in another (*n* = 1), and *Lodderomyces elongisporus* and *C. orthopsilosis* for the third patient (*n* = 1). One patient was co-colonised by *C. tropicalis* and *C. glabrata*. None of the colonised patients developed blood stream infections during hospitalisation.

### 3.2. Yeast Identification and Evaluation of CHROMagar^TM^ Candida Plus for C. auris

A total of 35 *C. auris* strains including 20 from 12 patients and 15 from environment samples were identified as *C. auris* by MALDI TOF-MS. *Candida auris* colonies were readily identified on selective CHROMagar^TM^ *Candida* Plus medium after 48 h of incubation showing distinct cream colour colonies with blue halo. In contrast, on CHROMagar *Candida* medium, *C. auris* was difficult to differentiate from other species that showed pink colonies which included *C. parapsilosis* (pink), *C. krusei* (pink fuzzy growth), *C. lusitaniae* (pink), *C. guilliermondii* (pink) and *Hyphopichia burtonii* (pink). Interestingly none of the above-mentioned non-*auris Candida* spp. showed colony morphology specific for *C. auris*, i.e., pale cream with distinctive blue halo on CHROMagar^TM^ *Candida* Plus medium [21].

### 3.3. Environmental C. auris

A total of 148 samples were collected from the environment of *C. auris* colonised patients during the study period. The results of environment sampling are detailed in Table 2. Overall, 10% (*n* = 15) of samples yielded *C. auris*. The maximum number of *C. auris* was recovered from floor (26.6%, *n* = 4) and bed railing (20%, *n* = 3), followed by patient bedside trollies (13.3%, *n* = 2). Further, *C. auris* was isolated from air conditioner air wings, bed sheet, pillow, mobile phone, and two medical equipment: oxygen mask and intravenous pole (IV) (Table 2). Out of 12 *C. auris* colonised patients, the environments of four patients (33.3%) were found to contain *C. auris* which included patients’ bed railing in three cases, followed by bed trolly (*n* = 2), and one case each of IV-pole, mobile phone of the patient, pillow, floor, and bed sheet. The environmental samples containing *C. auris* colonised patients became positive on an average of 8.5 days (duration 7–14 days) after patient’s colonisation was detected. Interestingly, environmental samples associated with the 20 non-*auris* yeast-colonised patients were all negative for *C. auris*. In addition, 13 yeast species other than *C. auris* were isolated from the environment screening specimens, including *C. parapsilosis*, *C. orthopsilosis*, *C. metapsilosis*, *C. guilliermondii*, *C. tropicalis*, *C. lusitaniae*, *C. albicans*, *C. catenulata*, *Pichia kudriavzevii, Trichosporon asahii*, *Hypopichia burtonii, Kodamea ohmeri*, and *L. elongisporus* (Table 2). Notably, pillow samples harboured the highest number of different yeast species (69%, 9 spp.), followed by floor (53.8%, 7 spp.), trollies (38.4%, 5 spp.), bed railing (30.7%, 4 spp.), sink (30.7%, 4 spp.), mobile (23%, 3 spp.), IV pole (23%, 3 spp.), bed sheet and air conditioner wings (15.3%, 2 spp.). Among these 13 species, five (*C. parapsilosis*, *C. orthopsilosis*, *C. guilliermondii, L. elongisporus,* and *C. tropicalis*) were found to colonise both patients and their environment.

### 3.4. Genome Sequencing and Phylogenetic Analysis of C. auris Isolates

**Figure 1:**
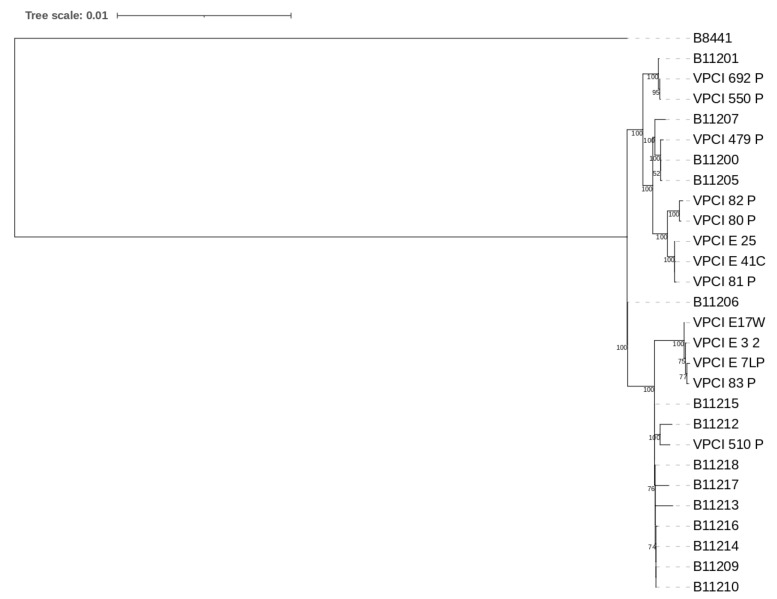
Maximum likelihood phylogenetic tree constructed based on 1000 bootstrap constructed by RAxML. Relationships among our nine strains and South Asian strains including 18 previously published Indian *C. auris* strains (B11200, B11201, B11205-B11207, B11209, B11210, B11212-B11218, VPCI_510/P/14, VPCI_692/P/12, VPCI_550/P/14, VPCI_479/P/13), and one reference Clade I strain B8441 from Pakistan.

A total of nine *C. auris* isolates representing clinical (*n* = 4), and environmental (*n* = 5) samples were analysed by whole genome sequencing. The four clinical isolates were from 3 patients (B, C, and D) and they were collected in the first four months of sampling. To explore the link of transmission from patient to environment and vice versa, five strains were chosen that represented environmental isolates related with the above three patients. Due to cost constraints, we were unable to sequence all *C. auris* isolates. All our nine isolates clustered in Clade I (South Asian clade) and showed an average SNP difference of 92 (minimum = 1, max = 160) among each other (Figure 1). Isolates varied from the Clade I reference strain B8441 (Pakistan, clade 1) by a minimum SNP difference of 900 and a maximum range up to 921. The nucleotide diversity (pi) [32] in the sample of all 28 South Asian Clade I isolates, including the 18 Clade I isolates from India that were retrieved from GenBank [25,26], was 1.36 × 10^−5^.

Phylogenetic analysis of these nine sequenced isolates (Figure 1) showed two closely related clusters (1 and 2) with 100% boot strap value. Cluster 1 comprised of one isolate of patient D (VPCI/83/P/2020) and three environmental isolates collected from vicinity of patient D in a single ward (VPCI/E/7LP/2020 from floor, VPCI/E/17W/2020 from patient′s mobile, VPCI/E/3/2020 from pillow). These isolates showed limited genetic difference among them with SNP difference of ≤8. Cluster 2 included five *C. auris* isolates which could be further divided into two subgroups. One subgroup includes two samples, VPCI/80/P/2020 (Ear; Patient B) and VPCI/82/P/2020 (Ear; Patient D), and they showed seven SNPs differences between each other. The other subgroup comprises three isolates, VPCI/E/41C/2020 (Bed railing of patient I), VPCI/E/25/2020 (Bed railing; Patient B), and VPCI/81/P/2020 (groin; Patient C) and showed ≤3 SNP differences between each other. A range of 43–136 SNPs differences were observed between *C. auris* isolates of the present study and previously published *C. auris* isolates from four different hospitals in India [25].

**Figure 2:**
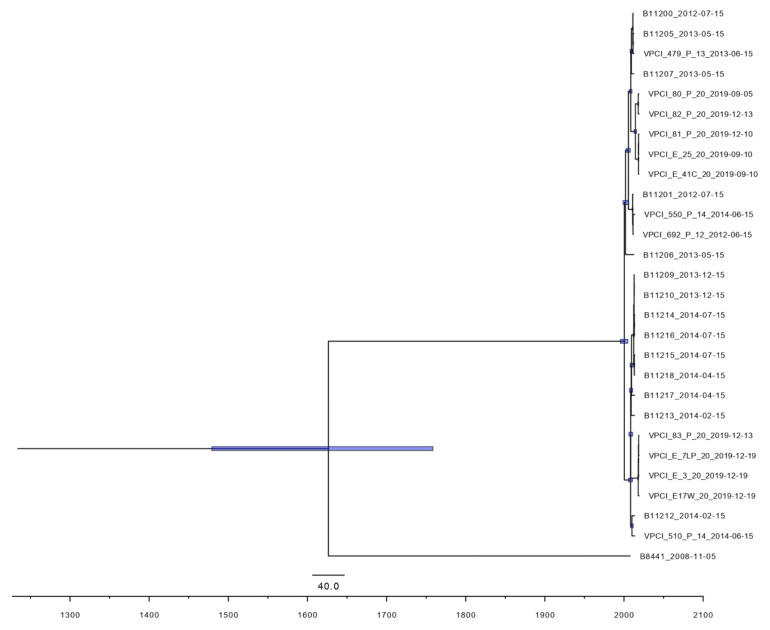
Maximum clade credibility phylogenetic tree of 9 *Candida auris* strains (clinical, *n* = 4; environmental, *n* = 5) isolated in the present study, and 18 previously published Indian *C. auris* strains, (B11200, B11201, B11205-B11207, B11209, B11210, B11212-B11218, VPCI_510/P/14, VPCI_692/P/12, VPCI_550/P/14, VPCI_479/P/13) along with Clade1 isolate (B8441) using BEAST strict clock model and coalescent model. Values indicate the posterior probability of the nodes in the maximum clade credibility tree. Purple bars indicate 95% highest posterior density.

To investigate the transmission patterns of *C. auris* strains colonizing patients and their respective environment, we derived the divergence times of the isolated strains and other Indian *C. auris* isolates from the same clade I based on the base substitution and divergence time framework reported previously [33]. According to the phylogeny, the most recent common ancestor for the clade I Indian strains dates back to the year 1626 (95% highest posterior density, 260.3–539.8 years ago). Consistent with the results of phylogenetic analysis, nine isolates of this study also grouped into two clusters according to their time of emergence (Figure 2). Cluster 1 had four isolates, one from ear of patient D (VPCI/83/P/2020), and three environmental isolates (VPCI/E/7LP/2020, VPCI/E/17W/2020 and VPCI/E/3/2020) from the vicinity of the same patient. The most recent common ancestor among these four isolates was estimated to have emerged in 2018 (95% HPD, 0.5–1.7 years ago). Cluster 2 included isolates from patients B, C, D along with environment isolates, VPCI/E/25/2020 (bed railing, patient B) and VPCI/E/41C/2020 (bed railing, patient I), and this cluster was estimated to have emerged about five years ago (95% HPD, 4.2–7.4 years ago). All the nine isolates were submitted to GenBank under accession number SAMN17149510-18.

### 3.5. Microsatellite Analysis

A total of 26 *C. auris* isolates comprising 16 from nine colonised patients (A–I) and 10 from their environmental samples were included for STR typing (Table 3). Overall, three distinct STR types (1, 2, and 3) were detected among 26 *C. auris* strains collected from nine patients and their environment. These three STR types were relatively equally represented, 10 isolates had STR 2 and eight each had STR 1 and STR 3. These three STR patterns differed in one or two loci (M3-1 FAM, M3-2 FAM). STR 1 strains were detected in two patients (A and D) and also from the environment of patient D, admitted in the same room. Interestingly, in patient D, different body sites i.e., ear, nose and groin were colonised by *C. auris* strains with three distinct STRs. Specifically, two different STRs i.e., 1 and 2, colonised the ear of patient D. Further, groin and nose were colonised by STR 1 and 3 respectively. Notably, the environment of patient D harboured STRs 1 and 2 on the personal mobile phone, pillow and floor samples. Notably, Patient I who was admitted after patient D, was assigned the same bed as previously occupied by patient D and, similar to patient D, developed colonisation with STR 3 in the nose. The remaining six patients (B, C, E–H) were admitted in four different rooms. All clinical and environmental isolates from these six patients harboured two STRs i.e., 2 and 3. Interestingly, the close environment of patient B had STR 3 on the bed railing. However, the patient was colonised with STR 2.

### 3.6. Comparison of Data Obtained with the STR Assay and WGS Analysis

*C. auris* strains that had identical STR varied by small SNP difference ranging 3–8 on WGS suggesting identical strains by both methods. Cluster 1 and two subgroups of cluster 2 identified by WGS represented three different STR types in each cluster/subgroup, suggesting concordant results by the microsatellite typing. WGS data of the three distinct genotypes detected in three patients (B, C, D) by STR analysis showed 30–150 SNP differences among each other. The isolates varied by one STR marker showed a SNP difference of 30 nucleotides whereas isolates varied by two STR markers showed a SNP difference of 150 nucleotides.

### 3.7. In Vitro Antifungal Susceptibility Testing (AFST) and Mutation Analysis

#### 3.7.1. Antifungal Susceptibility Testing

All of the 35 *C. auris* strains isolated from patients and their environment were subjected to in vitro antifungal susceptibility testing. MIC data against 10 antifungals tested are summarised in Table 4. All *C. auris* isolates in the present study were resistant to FLU (MICs ≥ 128 mg/L) barring one environmental isolate (MIC 16 mg/L) from the floor. Further, 25% clinical and 33% environmental *C. auris* isolates were non-susceptible to VRC (MIC ≥ 2 mg/L). Also 37% (*n* = 13) of isolates, including 23% (*n* = 8) clinical and 14% (*n* = 5) environmental, were resistant to AMB. Notably, 40% (*n* = 8/20) of clinical and 26.6% (*n* = 4/15) of environmental isolates were resistant to two classes of drugs (azoles+ AMB). Interestingly, *C. auris* isolates (VPCI/83/P/2020, VPCI/87/P/2020, VPCI/88/P/2020) from patient D and five isolates from the adjacent environment showed high MIC values of ≥2 mg/L against AMB. MFG and AFG showed MIC ranges below the proposed CDC tentative breakpoints (MIC ≥ 4 mg/L) for all isolates (Table 4).

#### 3.7.2. Genomic Analysis of Drug Resistant Genes

WGS analysis of nine isolates revealed the presence of two previously known amino acid substitutions associated with drug resistance, i.e., K143R (*n* = 5) or Y132F (*n* = 4) in the azole target *ERG11* gene in all *C. auris* isolates analysed, including an environmental isolate showing a low MIC of FLU (MIC 16 mg/L, Y132F) [34,35]. Further, analysis of the *TAC1B* gene, a zinc-cluster transcription factor-encoding gene, showed a previously described amino acid substitution A640V [36] in five of nine isolates. Notably, amino acid substitution A640V in *TAC1B* was exclusively observed in *C. auris* isolates that had K143R amino acid substitution in the azole target *ERG11* gene. Further, based on relative sequence read depth, five of nine (55.5%) *C. auris* isolates that harboured K143R amino acid substitution in the azole target *ERG11* gene likely had two copies of the *MDR1* gene (codes for a major facilitator transporter). Analysis of the ortholog of gene *YMC1* identified one amino acid substitution G145D present in isolates without the K143R amino acid substitution. Ortholog of *YMC1* has several transmembrane transporter activities and is essential in mitochondrial transport. Of note, all isolates have the (T > C) mutation in the 5′ end of this *YMC1* ortholog.

Analysis of other genes homologous to those involved in ergosterol biosynthesis in the Baker’s yeast *Saccharomyces cerevisiae* (*ERG2*, *ERG3*, *ERG5*, *ERG6*, *ERG20*, *ERG24*, *ERG25*, *ERG26*, *ERG27*, *ERG28*, and *ERG29*) revealed no amino acid substitutions relative to the reference strain B8441 in any of the nine strains, regardless of their susceptibility to AMB. Consistent with previous results showing all sequenced Clade I isolates having the *MTLa* locus, the mating type locus *MTLa* was found in all nine examined *C. auris* isolates. Also, all nine *C. auris* isolates analysed by WGS showed amino acid substitution K719N in the *STE6* gene, an ABC family transporter which is only expressed in *MTLa* carrying strains and exports the a-factor pheromone in *S. cerevisiae* and *C. albicans*, as required for mating [37]. 

## 4. Discussion

In this study, we report *C. auris* colonisation and transmission among patients with chronic respiratory diseases hospitalised in the medical wards of a chest hospital. *C. auris* colonisation occurred in one third (37.5%, 12 of 32 patients) of hospitalised patients. Notably, 9.3% of the screened patients (*n* = 3/32) were colonised at the time of admission suggesting prolonged carriage from outside of the hospital as a potential source of hospital contamination. In the present study, high rates of colonisation in chronic respiratory patients were recorded. It is known that the rates of culture positive surveillance cases of *C. auris* varies in different healthcare facilities depending on the facility type. Previously, a contact tracing and epidemiologic investigation surrounding cases earlier in the *C. auris* epidemic in New York revealed that colonisation rates varied by facility type, i.e., hospitals (5%), long term care facilities (LTCF, 6.3%), long term acute care (2.9%), and co-located hospital and LTCF (12.3%) [38]. *C. auris* colonisation rates in skilled nursing facilities that cared for ventilated patients were nearly ten times higher than the prevalence in skilled nursing facilities that did not provide care for ventilated residents [38,39]. It is pertinent to emphasize that colonisation by *C. auris* predisposes patients at risk for invasive infection as 5–10% of known colonised patients have been reported to develop invasive infections [10]. However, in the present study, none of the colonised patients developed invasive infections, probably attributed to the fact that none were on ventilatory support nor had invasive devices implanted. Both mechanical ventilation and invasive devices are considered major risk factors for the development of invasive infections due to *C. auris* [40,41]. Nevertheless, hospital environment contamination by the colonised patients could serve as a potential source for exogenous acquisition of multidrug-resistant *C. auris* [42]. Overall, 10% of environmental samples in the present study yielded *C. auris* including patients’ several contact objects, such as bed railings, bed sheets, pillows, bed side trolley and personal mobile phone. All of these could serve as the source of colonisation among the hospitalised population. A previous report from six US acute care hospitals in four states showed that *C. auris* was uncommon in hospital environments (3.8% of high-touch surfaces, 3.4% of sink drains, and 0% of portable equipment) in centers experiencing *C. auris* outbreaks during a six-month period [43].

To determine if environmental isolates of *C. auris* were genetically related to isolates that colonise patients, we undertook WGS and microsatellite genotyping. Interestingly, both techniques documented that multiple strains/genotypes contaminated the hospital environment and colonised patients. Further, we identified that genetically diverse strains colonised different body sites of patients. WGS identified close genetic relationships of strains (8–30 SNP variation) that colonised patients and their close environment probably due to shedding of the viable yeasts in the environment. Further, TMRCA analysis of an isolate from a single patient who developed colonisation after one week of admission suggested that the strain emerged prior to the study period and the patient probably acquired it from the environment. Interestingly, *C. auris* was not detected in the environment or rooms of patients who were not colonised by *C. auris* suggesting that the immediate environment is a potential source in acquiring *C. auris* in the hospital setting. Microsatellite length polymorphism (MLP) typing using short tandem repeats (STRs) has recently been validated for *C. auris* [18]. In the present study identical STR types differed by only 3–8 SNPs with WGS whereas different STR types exhibited 30–150 SNPs, suggesting concordant correlation among genomic analysis and STR genotyping. *C. auris* has a wide genetic diversity as evident by the previous genomic data analysis of 304 *C. auris* isolates from 19 countries. In addition, a wide genomic variability (SNP range, 43–136) among the *C. auris* population, circulating in different hospitals in India, was observed by WGS [33]. The present report extends the validation of STR typing in transmission settings and underscores the need of further analysis of this technique considering the wider genetic diversity of *C. auris* [44].

Identification of novel genetic determinants of antifungal susceptibilities significantly adds to the understanding of clinical antifungal resistance in *C. auris*. The genomic investigation of drug resistant genes implicated in fluconazole resistance in the present study showed the presence of either K143R or Y132F mutations in *ERG11* gene in clinical and environmental isolates of *C. auris*. Interestingly, a single *C. auris* isolate that had a low MIC of 16 mg/L for fluconazole also harboured Y132F amino acid substitution. This result suggests that other mechanisms also contribute to the development of fluconazole resistance. Also, 55% of *C. auris* isolates likely had two copies of the *MDR1* gene. A recent study analysed a global collection of *C. auris* isolates and demonstrated that a multitude of resistance-associated *TAC1B* mutations are present among the majority of fluconazole-resistant *C. auris* isolates specific to a subset of lineages or clades [36]. We observed that azole resistant *C. auris* isolates harboured amino acid substitution A640V (coexisting with K143R) in the zinc-cluster transcription factor-encoding gene *TAC1B*, suggesting that this mutation may provide additive fluconazole resistance in Indian *C. auris* isolates.

The molecular mechanisms of amphotericin B resistance and multiple drug resistance are poorly understood in *C. auris*. The present study reports high (37%) amphotericin B resistance in *C. auris* isolates colonizing patients and their environment. The acquisition of resistance to polyenes has been linked to mutations in the ergosterol biosynthesis pathway in *Candida* spp, including *ERG2* [45], *ERG6* [46], *ERG11* [47], and *ERG3* [48,49,50]. Recently, Ahmad et al., showed that a non-synonymous mutation in *ERG2* can lead to reduced susceptibility of amphotericin B in clinical *Candida glabrata* isolates [51]. However, no amino acid substitution was found in *ERG2* in any of our nine sequenced strains. The results suggest that resistance to amphotericin B is likely mediated by other genomic mutations. A recent report by Shivarathri et al. [52] described the role of the two-component signal transduction system and mitogen-activated protein kinase (MAPK) signalling pathway in drug resistance against AMB. The study demonstrated that genetic removal of *SSK1*, encoding a response regulator and the mitogen-associated protein kinase *HOG1*, restores the susceptibility to amphotericin B (AMB) in Indian *C. auris* clinical strains [52]. To tackle this yeast fundamental knowledge of mechanisms associated with amphotericin resistance and MDR in *C. auris* is warranted. Finally, to contain the spread of *C. auris* in healthcare settings, it is important to identify reservoirs and implement effective infection control practices [41]. During the early four-month-period of the study, routine cleaning and disinfection of patient rooms included use of a quaternary ammonium disinfectant once daily for disinfection of high-touch surfaces and a bleach wipe for post-discharge cleaning and disinfection. However, after the onset of the COVID 19 pandemic in late March in Delhi, India, the admission of chronic patients was limited. Further, COVID 19 pandemic control measures were placed stringently and enhanced cleaning procedures were implemented including using 1% sodium hypochlorite to clean and disinfect common surfaces 2–3 times a day. Resampling of *C. auris* positive environment in all the rooms showed clearance of *C. auris* from the environment, suggesting a high efficiency of 1% sodium hypochlorite against *C. auris* and a potential mechanism to prevent the transmission of this nosocomial yeast in a healthcare setting.

## Figures and Tables

**Table 1:** Details of 12 *Candida auris* colonised patients.

Patients Code	Age/Sex	Diagnosis	Weekly Culture Positivity (Body Sites Colonised)						Duration of Hospitalization
			1	2	3	4	5 *	On Discharge	
A	52/M	COPD, DM, HTN,	-	-	+ (G)	+ (G)		+ (G)	26 days
B #	60/M	COPD, Post tubercular cavity, DM, CPA	-	-	-	-	+ (E,N,G)	-	150 days
C #	64/M	COPD	-	-	+ (G)	+ (G)		+ (G)	25days
D #	47/M	COPD, post tuberculosis complications	-	-	+ (E,N,G)	+ (E,N,G)	-	-	58 days
E	52/M	HIV with pneumothorax	-	+ (G)	-	-	-	+ (G)	13 days
F	48/M	COPD, bronchiectasis	-	+ (E)				+ (E)	12 days
G	42/M	Post tuberculosis complications, cor pulmonale	-	-	+ (N)	+ (N)	-	-	33 days
H	45/M	COPD,	+ (N)	+ (N)				+ (N)	13 days
I	58/M	COPD, CPA	-	-	+ (E,N,G)	+ (E,N,G)	-	+ (E,N,G)	23 days
J	57/M	Post tuberculosis fibroatelectasis	-	-	+ (G)	+ (G)		+ (G)	24 days
K	51/F	ILD, DM	+ (G)	+ (G)	+ (G)			+ (G)	17 days
L	61/M	COPD, post tubercular cavity	+ (G)	+ (G)				+ (G)	10 days

- negative for *C. auris*. + positive for *C. auris*. * patients were screened every week till discharge (data after 5th week not included). # Patients isolates selected for whole genome sequencing. COPD, chronic obstructive pulmonary diseases; DM, diabetes mellitus; HTN, hypertension; CPA, cardiopulmonary arrest; ILD, interstitial lung diseases; E, ear; N, nose; G, groin.

**Table 2:** Distribution of yeast species isolated from patients’ environment samples.

Species (Number of Colonies)	Environment Sampling Sites (Number of Colonies)											
	Floor	Railing	Bed Sheet	Bed Side Trolley	IV Pole	Nebuliser	Oxygen Mask	A.C Wings	Pillow	Sink Samples	Mobile	Wheel Chair
*C. auris* (*n* = 15)	+ (*n* = 4)	+ (*n* = 3)	+ (*n* = 1)	+ (*n* = 2)	+ (*n* = 1)	-	+ (*n* = 1)	+ (*n* = 1)	+ (*n* = 1)	-	+ (*n* = 1)	-
*C. parapsilosis sensu stricto* (*n* = 75)	+ (*n* = 22)	+ (*n* = 13)	+ (*n* = 4)	+ (*n* = 5)	+ (*n* = 9)	+ (*n* = 1)	-	+ (*n* = 3)	+ (*n* = 13)	+ (*n* = 4)	+ (*n* = 1)	-
*C. orthopsilosis* (*n* = 4)	-	+ (*n* = 1)	-	+ (*n* = 1)	-	-	-	-	+ (*n* = 1)	-	+ (*n* = 1)	-
*C. metapsilosis* (*n* = 1)	-	-	-	-	-	-	-	-	+ (*n* = 1)	-	-	-
*C. guilliermondii* (*n* = 21)	+ (*n* = 5)	-	+ (*n* = 6)	+ (*n* = 2)	+ (*n* = 3)	-	-	-	+ (*n* = 1)	+ (*n* = 1)	-	+ (*n* = 3)
*C. tropicalis* (*n* = 11)	+ (*n* = 3)	+ (*n* = 1)	-	-	-	-	+ (*n* = 1)	+ (*n* = 2)	+ (*n* = 2)	+ (*n* = 1)	+ (*n* = 1)	-
*C. lusitaniae* (*n* = 5)	+ (*n* = 3)	-	-	+ (*n* = 2)	-	-	-	-	-	-	-	-
*L. elongisporus* (*n* = 3)	+ (*n* = 3)	-	-	-	-	-	-	-	-	-	-	-
*T. asahii* (*n* = 3)	+ (*n* = 2)	-	-	-	-	-	-	-	+ (*n* = 1)	-	-	-
*C. albicans* (*n* = 3)	-	-	-	+ (*n* = 1)	-	-	-	-	+ (*n* = 1)	+ (*n* = 1)	-	-
*H. burtonii* (*n* = 2)	-	-	-	-	+ (*n* = 2)	-	-	-	-	-	-	-
*K. ohmeri* (*n* = 2)	+ (*n* = 1)	+ (*n* = 1)	-	-	-	-	-	-	-	-	-	-
*P. kudriavzevii* (*n* = 1)	-	-	-	-	-	-	-	-	+ (*n* = 1)	-	-	-
*C. catenulata* (*n* = 1)	-	-	-	-	-	-	-	-	+ (*n* = 1)	-	-	-

+ Positive for respective yeast species. - Negative for respective yeast species.

**Table 3:** Short tandem repeat genotypes of 26 *Candida auris* isolates (patient body surface *n* = 16, patient environment *n* = 10) by using microsatellite typing using 12 STR markers.

Patient	Room No.	DOA/DOD	Patient Body Sites Positive for *C. auris* (STR Code)	Environment Sample Details (STR Code)	STR Code	M-2			M3-I			M3-II			M9		
						a	b	c	a	b	c	a	b	c	a	b	c
A	3	07-11-19/31-12-19	Groin (1)	Negative	1	66	19	9	60	10	18	37	29	22	19	11	9
B	3	01-08-19/30-12-19	Ear (2) *	Bed Railing (3) *	2	66	19	9	64	10	18	36	29	22	19	11	9
			Nose (2)		2	66	19	9	64	10	18	36	29	22	19	11	9
			Groin (2)		2	66	19	9	64	10	18	36	29	22	19	11	9
					3(BR)	66	19	9	62	10	18	36	29	22	19	11	9
C	1	21-11-19/17-12-19	Groin (3) *	Negative	3	66	19	9	62	10	18	36	29	22	19	11	9
D	4	01-12-19/28-01-20	Ear (1) *	Mobile (1) *	1	66	19	9	60	10	18	37	29	22	19	11	9
			Groin (1)	Pillow (1) *	1	66	19	9	60	10	18	37	29	22	19	11	9
			Ear (2) *	Floor ^1^ (1) *	2	66	19	9	64	10	18	36	29	22	19	11	9
			Nose (3)	Floor ^2^ (1)	3	66	19	9	62	10	18	36	29	22	19	11	9
				Floor ^3^ (1)	1(M)	66	19	9	60	10	18	37	29	22	19	11	9
				Floor ^4^ (2)	1(P)	66	19	9	60	10	18	37	29	22	19	11	9
					1(F ^1^)	66	19	9	60	10	18	37	29	22	19	11	9
					1(F ^2^)	66	19	9	60	10	18	37	29	22	19	11	9
					1(F ^3^)	66	19	9	60	10	18	37	29	22	19	11	9
					2(F ^4^)	66	19	9	64	10	18	36	29	22	19	11	9
E	2	15-01-20/28-01-20	Groin (2)	Negative	2	66	19	9	64	10	18	36	29	22	19	11	9
F	2	21-01-20/28-01-20	Ear (2)	Negative	2	66	19	9	64	10	18	36	29	22	19	11	9
G	2	03-01-20/06-02-20	Nose (2)	Negative	2	66	19	9	64	10	18	36	29	22	19	11	9
H	4	24-01-20/31-01-20	Nose (3)	Negative	3	66	19	9	62	10	18	36	29	22	19	11	9
I	4	07-01-20/30-01-20	Ear (2)	Oxygen mask (3)	2	66	19	9	64	10	18	36	29	22	19	11	9
			Groin (2)	Trolly (3)	2	66	19	9	64	10	18	36	29	22	19	11	9
			Nose (3)	Bed railing (3) *	3	66	19	9	62	10	18	36	29	22	19	11	9
					3(OM)	66	19	9	62	10	18	36	29	22	19	11	9
					3(T)	66	19	9	62	10	18	36	29	22	19	11	9
					3(BR)	66	19	9	62	10	18	36	29	22	19	11	9

Yellow, green and blue colours denote different STR genotypes, 1,2,3 respectively. DOA (Date of admission of patient), DOD (Date of discharge). * *C. auris* isolates selected for Whole genome sequencing. Env-Environment, BR-bed railing, M-mobile, P-pillow, F^1-4^-denotes different locations of the floor screened, OM-oxygen mask, T-trolly.

**Table 4:** MIC distribution of 35 *C. auris*, (patient body surface *n* = 20, patient environment *n* = 15) strains against 10 antifungal drugs tested using CLSI-BMD method.

Drugs ^a^	No. of Isolates with MIC/MEC (mg/L)														Range	GM ^b^	MIC_50_^c^
	≤0.015	0.03	0.06	0.125	0.25	0.5	1	2	4	8	16	32	64	≥128			
FLU												1	2	17	32–128	111.4	128
ITC					1		2	7	10						0.25–4	2.37	4
VRC					3	3	9	3	2						0.25–4	0.93	1
ISA	1		2	1	9	5	2								0.06–1	0.24	0.25
POS	1		1	5	9	4									0.015–0.5	0.19	0.25
AMB						12		8							0.5–2	0.87	0.5
5-FC			4	1	12	3									0.06–0.5	0.20	0.25
MFG			3	3	6	7	1								0.06–1	0.25	0.25
AFG				5	1	8	2	4							0.125–2	0.48	0.5
CFG					4		2	2	6	3					0.25–8	2.30	4
FLU											1		3	11	16–128	97	128
ITC							2	8	5						1–4	2.30	2
VRC					1	4	5	2	3						0.25–4	1.09	1
ISA		3	2	1	4	4	1								0.03–1	0.17	0.25
POS		3	1	3	8										0.03–0.25	0.13	0.25
AMB					4	5	1	2	3						0.25–4	0.74	0.5
5-FC		4	10	1											0.03–0.125	0.05	0.06
MFG		4	6	5											0.03–0.125	0.06	0.06
AFG				6	3	6									0.125–0.5	0.12	0.25
CFG					10	5									0.125–0.5	0.29	0.25

Shaded region indicates range of drug dilution of the corresponding drug. ^a^ FLC, fluconazole; ITC, itraconazole; VRC, voriconazole; ISA, isavuconazole; POS, posaconazole; AMB, amphotericin B; 5FC, flucytosine; AFG, anidulafungin; CFG, caspofungin; MFG, micafungin. ^b^ Geometric mean MICs, ^c^ MIC_50_, MIC at which 50% of test isolates were inhibited.

## Data Availability

All the nine isolates were submitted to GenBank under accession number SAMN17149510-18.
